# Supplementary material for: Molecular Approaches and Echocardiographic Deformation Imaging in Detecting Myocardial Fibrosis
Source: Int J Mol Sci. 2022 Sep 19;23(18):10944. doi: 10.3390/ijms231810944 (PMC9501415; doi:10.3390/ijms231810944)
Supplement: Supplementary file 1 [file ijms-23-10944-s001.zip › ijms-1867997-supplementary.pdf]

Supplemental Informations:

We searched PubMed, Embase and Scopus up to 30th June 2022. Eligibility criteria for study selection were the following: (1) reporting data with echocardiographic deformation imaging of the cardiac chambers; (2) myocardial fibrosis assessed by endomyocardial biopsy (EMB) and/or cardiac magnetic resonance imaging (CMRI); (3) biochemical and molecular measurements for the detection of myocardial fibrosis associated or not with deformation imaging parameters.

Supplemental Table

Supplemental Table S1 (ST1): Details of the included study on deformation imaging and their associations with invasive and non-invasive techniques in the assessment of myocardial fibrosis

Studies included	References
Strong inverse correlation between LV-GLS magnitude assessed by 2D-STE analysis and the extent of MF in EMB in patients with advanced heart failure, severe aortic stenosis, acute myocarditis and cardiac amyloidosis.	[74–82]
Strong negative correlation between LA reservoir strain assessed by 2D-STE analysis and the degree of LA fibrosis measured by EMB in patients with mitral valve disease and advanced heart failure.	[73,74,117]
Direct correlation of RV free wall myocardial deformation assessed by 2D-STE analysis with the extent of RV myocardial fibrosis by EMB in patients with end-stage heart failure	[120,121]
**Technical limitations of 2D-STE analysis**	
Long learning curve	[125]
Dependency on good image quality	[62]
Potential frame-to-frame decorrelation	[126]
The temporal stability of tracking patterns	[127]
The intervendor variability	[130,163]
Dependency on loading conditions (pre- and after-load), chamber geometry, dyssynchrony and segment interactions	[139,140]
The potential influence of chest wall conformation on myocardial strain parameters	[141–143,146]
**Biomarkers and Occurrence of myocardial fibrosis**	
Serum biomarkers occurrence	[27–29]
miR-21 occurrence	[46–51], [53–55], [76]
miR-185 occurrence	[56,57]
other miRs	[44,45]
